# Exploring the Efficacy of Methenamine Hippurate Across Different Patient Groups With Recurrent Urinary Tract Infections: Experience From a University Teaching Hospital in South Wales, United Kingdom

**DOI:** 10.7759/cureus.98511

**Published:** 2025-12-05

**Authors:** Philip Abolanle, Daniel Akintelure, Maike Eylert, Don S Wijayasuriya, Hasan A Al-Ibraheem, Coral Seymour, Paulette Hussain

**Affiliations:** 1 Urology Department, Royal Gwent Hospital, Aneurin Bevan University Health Board, Newport, GBR

**Keywords:** antibiotic prophylaxis, antimicrobial resistance, methenamine hippurate, recurrent uti, urogynaecology, uti prophylaxis

## Abstract

Introduction: Recurrent urinary tract infections (rUTIs) represent a significant clinical challenge, traditionally managed with long-term antibiotic prophylaxis. However, growing concerns regarding antimicrobial resistance have necessitated the exploration of alternative treatment modalities. Methenamine hippurate (MH), a urinary antiseptic with a unique mechanism of action, has emerged as a promising non-antibiotic option for rUTI prevention.

Objective: This study aims to examine the effectiveness of MH in the prophylaxis of rUTI, with a specific focus on patients with structural or functional abnormalities of the urinary tract and those requiring catheterization to empty their bladder.

Methods: A retrospective observational study was conducted on the first 150 patients prescribed MH in a University Hospital at a Local Health Board in South Wales from April 2020 to July 2022. Patient demographics, radiological findings, functional urological status, catheter use, and treatment outcomes were analyzed. Chi-square statistical tests were used to assess associations between categorical variables and treatment outcomes.

Results: The cohort comprised 132 females (88%) with a median age of 60 years. After 12 weeks of treatment, 100 (66.7%) of patients showed improvement, with about half of these (n = 46) experiencing complete resolution of UTIs and a further third (n = 35) reporting reduced frequency or severity at six months follow-up. No statistically significant difference in long-term outcomes was observed based on radiological findings (χ^2^ = 0.138, df = 2, p = 0.933). Similarly, there was no significant association between functional urological status and treatment outcome (χ^2^ = 4.763, df = 2, p = 0.092), nor between catheter use and outcome (χ^2^ = 4.226, df = 2, p = 0.121)

Conclusions: MH demonstrates effectiveness for long-term management of rUTIs across diverse patient populations, including those with structural or functional urinary tract abnormalities and catheter users. These findings support the broader application of MH as a viable alternative to antibiotic prophylaxis, potentially reducing antimicrobial resistance while maintaining clinical efficacy.

## Introduction

Recurrent urinary tract infections (rUTIs) are defined as two or more episodes within six months or three or more episodes within one year [1]. This condition predominantly affects women, with an annual incidence of approximately 30 per 1,000 women [2]. The burden of rUTIs extends beyond acute symptoms such as dysuria, urgency, and suprapubic pain, with potential complications including renal damage, sepsis, and reduced quality of life [3].

Traditionally, continuous antibiotic prophylaxis has been the mainstay of rUTI management. However, this modality carries substantial risks, including the development of antimicrobial resistance, alteration of normal flora, and potential adverse effects from prolonged antibiotic exposure [4,5]. Shorter inter-infection intervals were significantly associated with elevated antimicrobial resistance, particularly among patients undergoing repeated antibiotic courses. Notably, resistance frequently encompassed multiple antimicrobial classes [6]. The UK Antimicrobial Resistance Strategy has emphasized the urgent need for antimicrobial stewardship and alternative prevention strategies [7].

Methenamine hippurate (MH) is a promising non-antibiotic option for preventing rUTIs. MH undergoes hydrolysis in acidic urine (pH <5.5), producing formaldehyde and ammonia. Formaldehyde acts as a broad-spectrum antimicrobial by denaturing bacterial proteins and nucleic acids [8]. The hippuric acid component maintains urinary pH acidity, creating an unfavourable environment for bacterial growth while enhancing formaldehyde formation. Notably, MH has minimal systemic metabolism and a half-life of approximately four hours [8].

Recent landmark trials have generated strong evidence supporting the efficacy of MH in preventing rUTIs. The ALTAR trial, a large randomized pragmatic study, demonstrated that MH is non-inferior to daily low-dose antibiotics in preventing rUTIs in women, while showing lower rates of antimicrobial resistance [9]. A Cochrane systematic review suggested that MH may be particularly effective in patients without renal tract abnormalities [10], though data in patients with structural or functional abnormalities remains limited.

The ImpresU trial, a triple-blind randomized controlled trial in older women, found that MH reduced rUTI frequency by an estimated 25% compared to placebo, with mild side effects and low potential for antimicrobial resistance selection [11]. However, discontinuation after six months appeared to increase urinary tract infection (UTI) relapse risk. Cost-effectiveness analyses indicate that while antimicrobial prophylaxis may be less expensive initially, MH offers a valuable alternative due to its potential to reduce antimicrobial use and mitigate associated public health risks [12,13].

Despite this growing evidence base, several knowledge gaps remain. Most studies have focused on women with uncomplicated rUTIs, leaving uncertainty regarding MH effectiveness in patients with structural urinary tract abnormalities, functional disorders, or those requiring catheterization. The National Institute for Health and Care Excellence (NICE) has highlighted the need for research examining MH effectiveness in men, pregnant women, children, and patients with complicated UTIs [14]. These complex rUTI subgroups are clinically important because they experience higher recurrence rates, antimicrobial resistance, and limited response to conventional prophylaxis. Highlighting these populations emphasizes the potential role of MH as a non‑antibiotic preventive strategy.

This study aims to evaluate experiential outcomes of MH prophylaxis across diverse patient populations within a university teaching hospital, with a specific focus on the impact of structural abnormalities, functional impairments, and catheter use on treatment response.

## Materials and methods

Study design and setting

This retrospective observational study was conducted at a university teaching hospital within a Local Health Board in South Wales, United Kingdom. The analysis included the first 150 consecutive patients prescribed MH between April 2020 and July 2022.

Patient selection

All patients prescribed MH during the study period were included. The indication for treatment was rUTIs, defined as two or more symptomatic episodes within six months or three or more within one year. All cases of rUTI included in this study were confirmed by positive urine cultures in the presence of compatible clinical symptoms.

Treatment protocol

All patients initially received MH at a dose of 1g twice daily for three months and were assessed afterward. Patients were offered a further three-month course if they wished to continue. At the end of the six-month course, patients who responded well to treatment were discharged from the Urology service to continue treatment with their general practitioners (GPs), as MH was later added to the formulary for GPs to prescribe. Non-responders were referred to specialist clinics for consideration of other treatment options.

Data collection

Data were retrospectively collected from available hospital electronic medical records. Variables extracted included demographics, radiological assessment, functional urological status, cystoscopy findings (when performed), catheter use, treatment outcomes (initially at 12 weeks and subsequently at six months and beyond), side effects, and tolerability. Treatment adherence was assessed through patient self‑report during follow‑up consultations. Side effects were monitored through spontaneous patient reports during clinic visits and review of consult notes; no structured adverse event interviews were performed.

Outcome measures

Primary outcomes were categorized as complete resolution (no further culture‑confirmed UTIs with absence of symptoms), partial improvement (a reduction in the frequency and/or severity of culture‑confirmed infections compared with baseline), or no improvement (persistent UTIs with unchanged frequency and severity). Outcome classification was based on patient‑reported symptoms corroborated by available culture results.

Secondary outcomes included treatment tolerability, adverse events, and reasons for discontinuation. Safety was assessed by patient‑reported side effects and documentation of therapy cessation. Additional secondary measures were subgroup comparisons (structural and functional abnormalities, catheter use, and sex) to evaluate whether MH effectiveness varied across different patient populations.

Statistical analysis

Descriptive statistics were used to summarize patient demographics and treatment outcomes. Associations between categorical variables, including radiological findings, functional status, catheter use, and cystoscopy findings, and treatment outcomes were assessed using chi-square tests. A p-value of <0.05 was considered statistically significant. All analyses were conducted using appropriate statistical software. Specifically, we used IBM SPSS Statistics for Windows, Version 27 (Released 2019; IBM Corp., Armonk, New York, United States). Missing data were excluded from the analysis, and all reported percentages and statistical tests were based on available cases only.

Ethical considerations

This retrospective study was based on routinely collected clinical data. Patient confidentiality was maintained throughout, with all data anonymized prior to analysis.

## Results

Patient demographics and baseline characteristics

A total of 150 patients were included in the analysis. The cohort was predominantly female (132 patients, 88%), with 18 male patients (12%). The median age was 60 years (mean 57 years; range: 19-87 years). Radiological assessment of the urinary tract revealed normal findings in 121 patients (80.7%) and abnormal findings in 29 patients (19.3%). Abnormal findings encompassed structural and post‑surgical changes such as kidney stones, prior pyeloplasty, long‑standing hydronephrosis, reimplanted ureters, Mitrofanoff, and transplanted kidneys. Functional urological status was normal in 103 patients (68.7%) and abnormal in 47 patients (31.3%). Functional abnormalities included detrusor overactivity, neurogenic bladder, Fowler’s syndrome, and bladder pain syndrome.

Cystoscopy was performed in 76 patients (50.6%), with normal findings in 59 (39.3%) cases. The most common abnormal findings were cystitis cystica and bladder trabeculations. Twenty-eight patients (18.7%) used catheters for bladder emptying: 22 (14.7%) performed intermittent self-catheterization (ISC), four (2.7%) had suprapubic catheters (SPC), and two (1.3%) had indwelling urethral catheters.

Treatment outcomes

Initial Outcomes (12 Weeks)

Following the initial 12-week MH regimen, 100 patients (66.7%) experienced clinical improvement. This included patients who either had no further UTIs or reported reduced frequency or severity of infections. Twenty-four patients (16.0%) showed no improvement, while outcomes were unclear in 26 patients (17.3%) due to treatment discontinuation related to side effects or loss to follow-up. Among evaluable patients (n = 124), the overall likelihood of improvement was high (80.6%), with patients over four times more likely to improve than not improve (odds ratio (OR) 4.17, 95% confidence interval (CI): 2.39-7.28; risk ratio (RR) 4.17, 95% CI 2.39-7.28).

Long-Term Outcomes (Six Months and Beyond)

At six-month follow-up, 46 patients (30.7%) maintained clinical improvement with no further UTIs. An additional 35 patients (23.3%) continued to experience UTIs, but with reduced frequency or severity, or reported subjective improvement and expressed willingness to continue treatment. Six patients (4.0%) continued to show no clinical improvement, and 15 patients (10%) had transitioned to alternative therapies, most commonly intravesical hyaluronic acid (iAluril). One patient was subsequently prescribed low-dose antibiotic prophylaxis. Of the remaining 47 patients, 24 demonstrated initial improvement and were discharged from the Urology service, while 23 were lost to follow-up. Among evaluable patients, the overall likelihood of sustained or partial improvement was high (n = 81, 54.0%), with patients more than twice as likely to demonstrate benefit than to show no improvement (OR 2.25, 95% CI 1.02-4.95; RR 2.25, 95% CI 1.02-4.95).

Outcomes in Special Populations

Male patients: Among the 18 male patients in the cohort, 14 (77.7%) had normal urinary tract imaging, while 11 (61.1%) exhibited functional urological abnormalities. Following the initial three-month MH course, 15 men (83.3%) demonstrated clinical improvement. At the six-month follow-up, 12 men (66.7%) maintained sustained improvement, with fewer UTI episodes. Overall, male patients were more likely to improve compared with female patients (83.3% vs. 64.4%; OR 2.83, 95% CI 0.78-10.26; RR 1.29, 95% CI 0.98-1.70), though subgroup size was small and CIs were wide.

Catheter users: Among the 28 catheter users, the overall success rate was 64% (n = 18).

Radiological status and treatment outcomes

**Figure 1:**
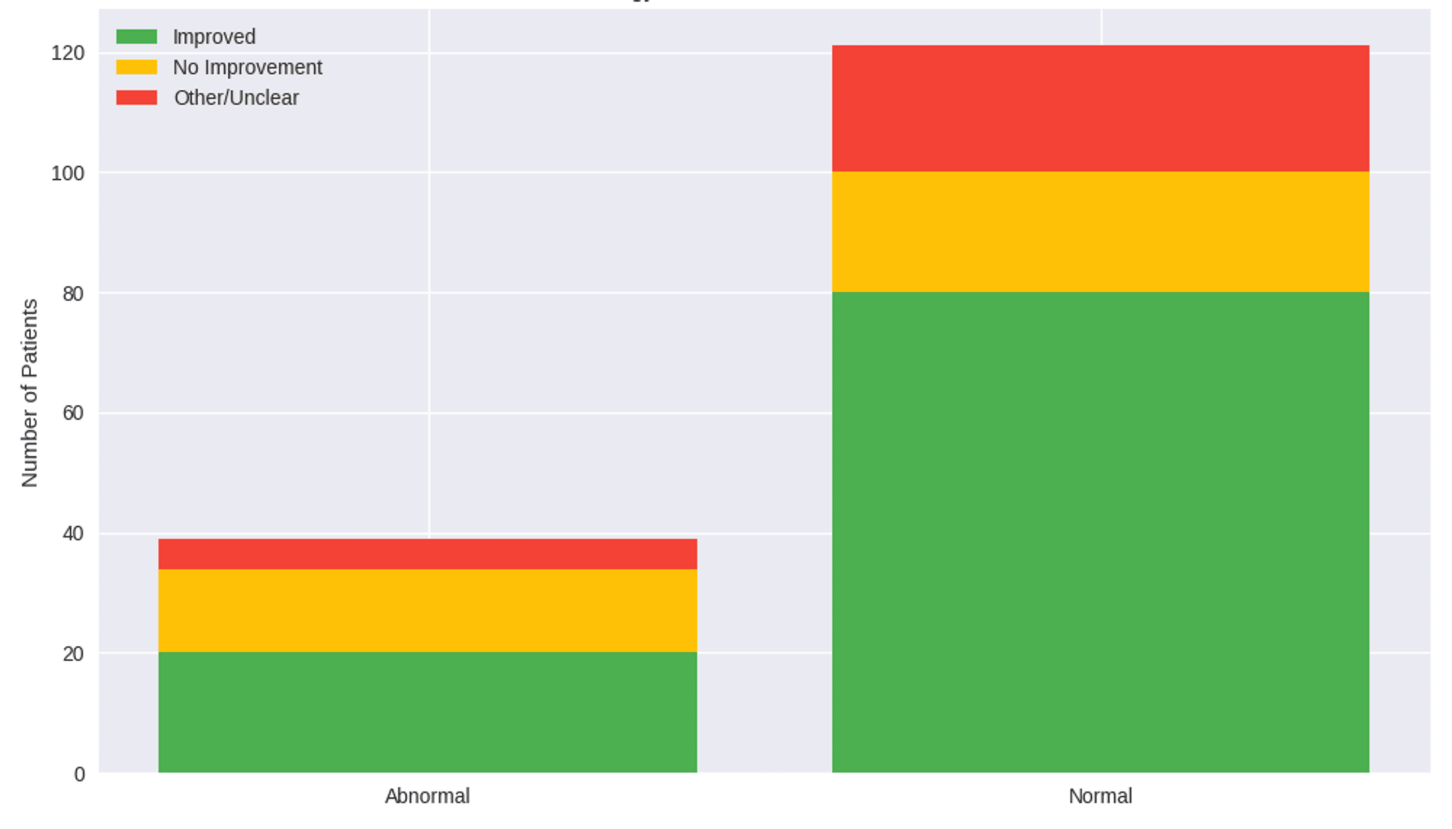
Clinical outcomes following MH therapy according to radiological findings MH: methenamine hippurate

Patients with abnormal radiological findings demonstrated a comparable rate of clinical improvement (n = 20, 69.0%) to those with normal imaging (n = 80, 66.1%). This difference was not statistically significant (χ^2^ = 0.138, df = 2, p = 0.933), indicating that radiological status did not significantly influence initial treatment outcomes (Figure 1). Effect size estimates confirmed this, with similar odds and relative risks of improvement between groups (OR 0.89, 95% CI 0.37-2.14; RR 0.96, 95% CI 0.72-1.28).

Functional status and treatment outcomes

**Figure 2:**
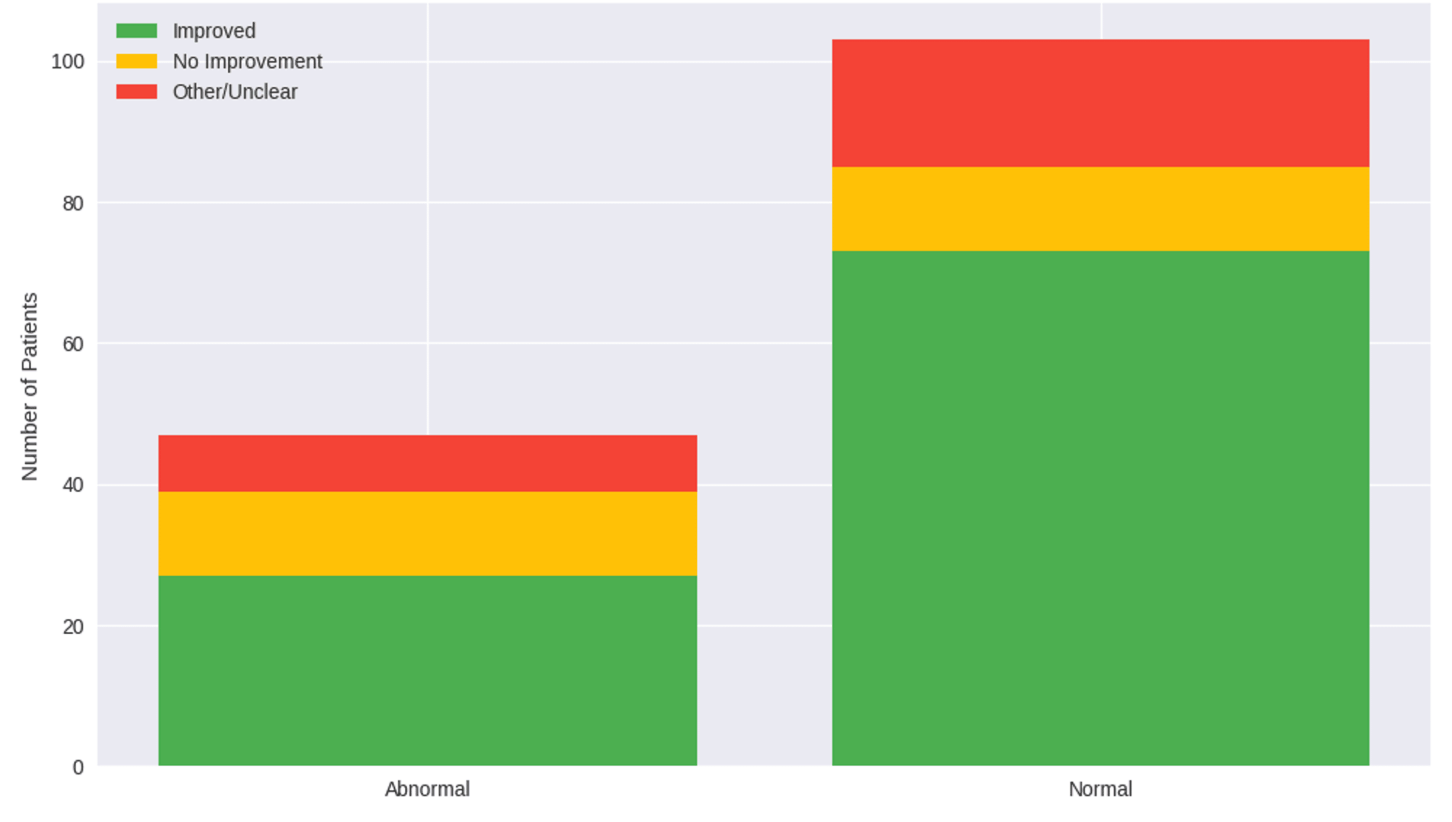
Outcomes of treatment with MH based on functional urological status MH: methenamine hippurate

Patients with normal functional urological status demonstrated higher initial improvement rates (n = 73, 70.9%) compared to those with functional abnormalities (n = 27, 57.4%). However, this difference did not reach statistical significance (χ^2^ = 4.763, df = 2, p = 0.092) (Figure 2). Effect size estimates suggested a non‑significant trend toward benefit in patients with normal function (OR 1.82, 95% CI 0.91-3.65; RR 1.23, 95% CI 0.94-1.62).

Catheter use and treatment outcomes

**Figure 3:**
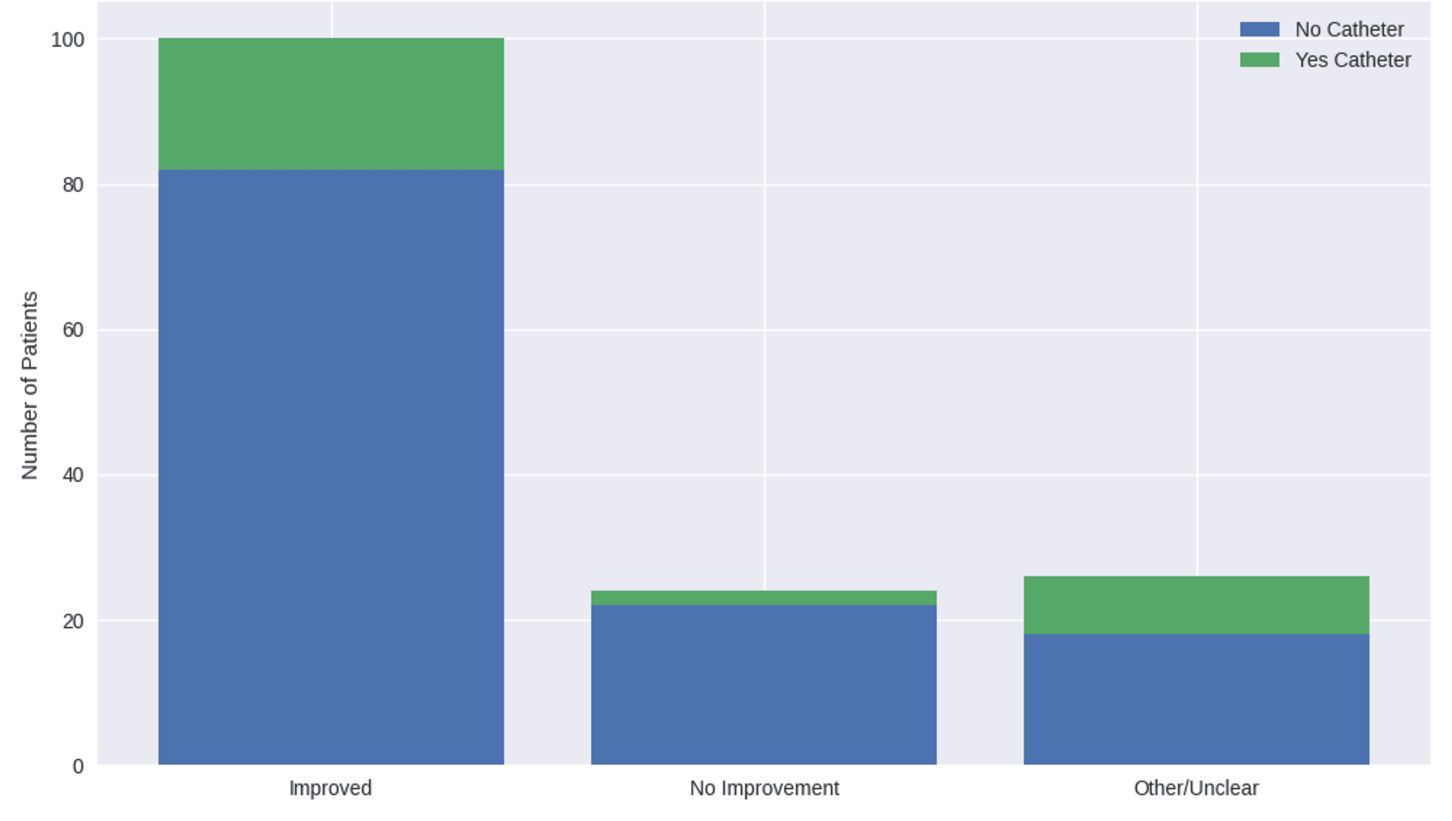
Outcome of MH treatment based on the patient's use of catheter MH: methenamine hippurate

Patients who used catheters demonstrated improvement rates (64.3%) comparable to those who did not require catheterization (67.2%). This difference was not statistically significant (χ^2^ = 4.226, df = 2, p = 0.121), indicating that catheter use did not significantly influence treatment outcomes (Figure 3). Effect size estimates confirmed this finding, with comparable odds and relative risks of improvement between groups (OR 0.88, 95% CI 0.39-1.99; RR 0.96, 95% CI 0.67-1.37).

Safety and tolerability

The majority of patients (n = 107, 71.3%) reported no side effects during MH treatment. Twenty-two patients (14.7%) experienced side effects or treatment intolerance, with nausea being the most frequently reported specific complaint. Overall, the adverse event rate was low, and MH was generally well tolerated.

## Discussion

This observational study supports the effectiveness of MH as a prophylactic agent for rUTIs across a clinically diverse patient population, including individuals with structural abnormalities, functional impairments, and catheter dependence. These findings are consistent with existing literature and extend the evidence base by offering valuable insights into MH use among patient groups historically underrepresented in clinical trials.

Efficacy in the general population

Our overall improvement rate of 66.7% at 12 weeks is consistent with findings from recent large-scale studies. The ALTAR trial demonstrated the non-inferiority of MH compared to daily antibiotic prophylaxis in women with rUTIs [9], while Botros et al. reported similar recurrence rates between MH and trimethoprim (p = 0.98) [15]. Our sustained improvement rate of 30.7% at six months, defined by complete resolution of UTIs, further supports the long-term efficacy of MH. This rate, although lower than some previously reported outcomes, may reflect the inclusion of a more complex patient population, including individuals with structural or functional abnormalities and catheter dependence.

The ImpresU trial in older women reported a 25% reduction in rUTI frequency with MH [11], which is comparable to our observed outcomes. Notably, that study also documented increased UTI relapse following treatment discontinuation, underscoring the importance of sustained prophylaxis in susceptible populations.

Novel findings in special populations

Structural and Functional Abnormalities

A key finding of this study is the comparable efficacy of MH regardless of structural or functional urinary tract status. The Cochrane review by Lee et al. reported no benefit of MH in patients with renal tract abnormalities (symptomatic UTI: RR 1.54, 95% CI 0.38-6.20) [10], suggesting limited utility in this subgroup. In contrast, our data showed no statistically significant difference in outcomes between patients with normal (66.1% improvement) and abnormal (69.0% improvement) radiological findings (p = 0.933). This discrepancy may reflect differences in study design, patient selection, or the operational definition of “renal tract abnormalities.” These findings suggest that MH should not be routinely excluded from consideration in patients with structural abnormalities, although prospective studies are warranted to validate this observation.

Catheter Users

Our finding that catheter use did not significantly affect MH efficacy (p = 0.257) is particularly noteworthy. The Cochrane review by Lee et al. suggested a limited benefit of MH in patients with neuropathic bladder or catheter dependence [10]. In contrast, Wade et al. reported variable success rates among catheter users, with 50% improvement in those with indwelling catheters (urethral or suprapubic) and 67% in patients using intermittent catheterization [16]. Our results align more closely with Wade’s findings, demonstrating a 64% overall improvement rate in catheter users. The mechanism underlying MH efficacy in catheterized patients warrants further investigation. Continuous urinary drainage may reduce stasis and enhance the antiseptic activity of formaldehyde.

Male Patients

Although small (n = 18), our male cohort demonstrated encouraging results, with 83.3% showing initial improvement and 66.7% maintaining sustained benefit at six months. This is notable given the NICE evidence review highlighted a lack of data regarding MH efficacy in men with rUTIs [14]. While the limited sample size precludes definitive conclusions, these findings suggest that MH may be a viable prophylactic option for male patients - a subgroup warranting further prospective investigation.

Safety and tolerability

The low adverse event rate observed in our study (14.7% reporting any side effects) is consistent with existing literature. Davidson et al.'s systematic review found that adverse effects associated with MH were comparable to those of other prophylactic agents, most commonly including nausea, abdominal pain, and headache [17]. Similarly, the ImpresU trial reported only mild side effects [11], further supporting MH’s favourable safety profile relative to long-term antibiotic prophylaxis.

Antimicrobial stewardship implications

The antimicrobial stewardship implications of MH are substantial. The ALTAR trial demonstrated proportionally higher antimicrobial resistance in women taking prophylactic antibiotics compared to those on MH [9]. Beerepoot et al. found that, unlike trimethoprim-sulfamethoxazole, lactobacilli (another non-antibiotic alternative) did not increase antibiotic resistance, though they failed to meet non-inferiority criteria [18]. MH's mechanism of action through formaldehyde generation provides a non-resistance-inducing alternative that aligns with current antimicrobial stewardship priorities [7].

Recent expert reviews have highlighted MH's resurgence as an important strategy in the prevention of rUTIs, particularly in the context of growing antimicrobial resistance [19,20]. While cost analyses suggest antimicrobial options may be cheaper in the short term [13], the broader public health benefits of reduced resistance and preservation of antibiotic effectiveness make MH an increasingly valuable therapeutic option [12]. If no benefit is observed at six months despite appropriate urinary acidification and addressing modifiable factors, MH should be discontinued [8].

Limitations

This study has various limitations inherent to its retrospective design, including the potential for confounding that cannot be fully controlled. The high proportion of unclear outcomes reflects common challenges in routine clinical practice, such as incomplete follow‑up and variability in data capture. Loss to follow‑up may have introduced bias, as outcomes in these individuals remain unknown. Handling of missing data is another limitation; outcomes were reported only for patients with available follow‑up, and missing data were not imputed. The relatively small sample sizes in certain subgroups - particularly male patients and specific catheter types - reduced statistical power to detect subgroup differences. Functional abnormalities were assessed clinically in many cases, which may have introduced classification variability. In addition, the study period coincided with the COVID‑19 pandemic, which restricted routine clinical reviews and limited opportunities for culture confirmation, thereby contributing to incomplete data capture. These constraints highlight the need for prospective, controlled studies with standardized data collection to confirm and extend our findings. Despite these limitations, this study provides valuable real‑world insights into the use of MH in complex rUTI populations and highlights its potential contribution to antimicrobial stewardship.

## Conclusions

This observational study demonstrates that MH is an effective prophylactic agent for rUTIs across a diverse patient population. Treatment efficacy was comparable regardless of structural abnormalities, functional status, or catheter use, challenging prior assumptions about MH limitations in complex clinical subgroups. With two-thirds of patients showing improvement at 12 weeks and nearly one-third maintaining complete resolution at six months - alongside a low adverse event rate - MH represents a viable non-antibiotic alternative to long-term antimicrobial prophylaxis.

In the context of rising antimicrobial resistance and the need for stewardship, MH offers a valuable option that warrants broader consideration in clinical practice. These findings support expanding MH use beyond its traditional indication in women with uncomplicated rUTIs to include patients with structural and functional abnormalities, catheter dependence, and male patients. While prospective studies are needed to confirm these results, MH should be considered early in the therapeutic algorithm for rUTI prevention, particularly in patients at risk of resistance or with contraindications to antibiotic therapy.
